# Non-Destructive Prediction of NaCl Content in Pork During Ultrasound-Assisted Marination: Multiphysics Simulation and Electrical Impedance Spectroscopy

**DOI:** 10.3390/foods15111976

**Published:** 2026-06-02

**Authors:** Lina Guo, Xin Ling, Mengyue Lu, Chen Hong, Xinyan Zhang, Ningning Ouyang, Hui Luo, Haile Ma

**Affiliations:** 1 School of Food Science and Engineering, Jiangsu University, No. 301 Xuefu Road, Zhenjiang 212013, China; guolina@ujs.edu.cn (L.G.);; 2 Institute of Food Physical Processing, Jiangsu University, No. 301 Xuefu Road, Zhenjiang 212013, China; 3 Key Laboratory of Zhenjiang, Institute for Energy Research, Jiangsu University, Zhenjiang 212013, China; 4 Taizhou Key Laboratory of Food Biomanufacturing, School of Food Science and Health, Jiangsu Agri-Animal Husbandry Vocational College, Taizhou 225300, China

**Keywords:** ultrasound-assisted marination, NaCl diffusion, visualization, multiphysics simulation, electrical impedance

## Abstract

This study investigated the effects of ultrasound-assisted marination on NaCl diffusion in pork using multiphysics simulation and evaluated the accuracy of electrical impedance spectroscopy for predicting NaCl content during marination. The results showed that short-term ultrasonic treatment did not significantly enhance moisture diffusion from brine into pork tissue. However, multiphysics simulation demonstrated that ultrasound significantly accelerated NaCl penetration, enabling a reduced brine concentration without compromising the final salt content, as further confirmed by thermogravimetric analysis, which showed higher residual NaCl and mass in treated samples. Electrical impedance properties exhibited systematic changes with increasing ultrasonic marination time, including decreased impedance, increased phase angle, and a reduced Cole–Cole arc radius, reflecting enhanced NaCl diffusion and structural modifications in muscle tissue. A strong linear correlation between impedance parameters and NaCl content was established, and validation results confirmed that impedance spectroscopy can accurately predict NaCl levels during marination. These findings highlight the potential of combining ultrasound-assisted marination with impedance-based techniques for real-time, non-destructive monitoring of salt content in meat processing.

## 1. Introduction

Meat is a cornerstone of the human diet due to its high nutritional value, containing essential amino acids, vitamins, minerals, and fatty acids [1]. These nutrients are necessary for proper growth, development, and human evolution, and they are difficult to obtain from other foods [2]. Meat is also prone to spoilage; therefore, the fundamental technology of preservation by curing was discovered, which reduced water activity and provided antimicrobial resistance, thereby extending shelf life [2,3]. Nowadays, curing is not merely employed for meat preservation but also serves as a preconditioning technique preceding cooking, intended to enhance the flavor and texture of the meat [4]. Traditional marination methods under ambient conditions are time-consuming and lead to deterioration in meat quality, particularly during hot summer months [5]. Consequently, consumers have increasingly adopted refrigerator-based low-temperature preconditioning marination approaches. However, traditional low-temperature marination faces greater challenges, including the slow mass transfer of marinade and limitations in penetration depths, which cause a waste of time and uneven distribution of salt inside and outside the meat, thereby failing to meet the demands of consumers for a fast-paced lifestyle and nutritious, healthy diets [6]. Therefore, the development of innovative, rapid-marination technologies that enhance meat-curing efficiency and quality has emerged as a research priority [7].

Ultrasound is a novel physical processing technology that is widely used in the fields of meat freezing [8], thawing [9], drying [10], tenderization [11], and marination [12], which can be attributed to its potent penetrative force and good practicability [13]. Numerous studies have demonstrated that ultrasonic marination significantly accelerates the mass transfer and improves meat quality through several ultrasonic mechanisms, including cavitation, mechanical effects, and the “sponge effect” [14,15]. Currently, there is limited availability of ultrasonic equipment specifically designed for low-temperature meat marination in home kitchens. Most studies have employed probe-type ultrasonic systems, which often lead to uneven acoustic distribution, with highly concentrated energy near the probe and localized overheating of the meat [16,17,18]. In contrast, bath-type ultrasound systems can provide a more uniform acoustic field and help avoid localized overheating. Furthermore, the effectiveness of ultrasonic marination is jointly governed by several processing parameters, including ultrasonic frequency, ultrasonic intensity, and treatment time. Among these, ultrasonic frequency is considered a critical factor because it directly affects cavitation characteristics, energy dissipation behavior, and the extent of structural perturbation induced in meat tissues [19]. Variations in frequency may therefore lead to substantial differences in mass transfer efficiency, protein conformational changes, water distribution, and tissue microstructure, ultimately affecting marination rate, tenderness, water-holding capacity, and overall product quality [20]. In particular, low-frequency ultrasound is generally associated with stronger cavitation and enhanced penetration of marinating agents, whereas high-frequency ultrasound may provide a more uniform but milder processing effect. Therefore, elucidating the role of ultrasonic frequency in marination is essential for understanding the underlying mechanism of ultrasound-assisted marination and for optimizing the process to improve product quality.

In meat marination, sodium chloride (NaCl) is an essential ingredient because it imparts a characteristic salty taste and improves tenderness and flavor. However, over-intake of NaCl will increase the risk of hypertension and cardiovascular disorders, mainly due to the adverse effects of sodium [21]. Hence, accurate detection of NaCl content is important during the marination process of meat. Currently, the determination of NaCl content predominantly relies on the traditional hydrolysis titration method, an offline chemical detection technique [22]. However, this method is limited by its labor-intensive and time-consuming nature, as well as its heavy reliance on chemical reagents, leading to environmental issues [23]. Hence, it is necessary to introduce a novel, rapid, and efficient non-destructive testing technology to monitor the NaCl content in real-time during the curing process. This will ultimately provide intelligent technical support for industrial meat marination.

Electrical impedance spectroscopy (EIS) is an emerging technology that applies a small-amplitude periodic disturbance signal to a sample without compromising its stability and then utilizes the linear relationship between the sample’s response and the excitation signal at various electrical frequencies to obtain its internal information [24]. EIS in biological tissues mainly includes three important fundamental theories: Schwan dispersion theory, Cole–Cole theory, and Fricke’s equivalent circuit model [25]. The phenomenon where the impedance of biological tissues undergoes drastic changes within a certain frequency range is called frequency scattering, or simply dispersion [26]. Schwan proposed that there are three main scattering distributions of biological tissues in different frequency ranges, namely α dispersion, β dispersion, and γ dispersion [27]. α and β dispersion can better reflect the state of cells than γ dispersion and are often used in impedance measurement in biological tissue research. The Cole–Cole model reflects the changes in cell structure by describing the electrical properties of biological tissues. The Fricke model is a method that uses electronic components to simulate biological systems at the microscopic level. It holds that biological tissues are uniform suspensions of cells in an electrolyte liquid medium. EIS has been widely used in the field of food engineering, owing to its sensitivity to structural and compositional changes in biological tissues. It has been extensively applied to monitor physicochemical modifications induced by processing operations, including drying and freezing-thawing treatments in eggplant pulp [28] and texture degradation in thermosonicated carrot tissue [29]. Moreover, EIS has demonstrated considerable potential for quality evaluation, such as freshness assessment of carp [30], quantification of salt and water content in fish [31], and characterization of microstructural alterations in pork during storage [32]. More recently, it has also been explored as a predictive tool for assessing the textural properties of complex meat systems, including *Tricholoma matsutake* [33], emulsified sausages [34], and beef [35]. Despite the growing application of EIS in food systems, its use for quantitatively tracking NaCl dynamics during marination remains limited. In particular, the lack of mechanistic understanding linking impedance responses to salt diffusion and tissue structural evolution under ultrasound-assisted conditions restricts its broader application as a reliable predictive tool.

The present study aims to develop a non-destructive and mechanism-informed framework for predicting NaCl content during ultrasound-assisted marination. Specifically, multiphysics simulations based on COMSOL 5.2 are employed to visualize NaCl distribution in pork under varying ultrasonic frequencies, followed by thermogravimetric analysis to validate the simulation accuracy. EIS equivalent circuit modeling is then applied to characterize microstructural changes in tissue, and a quantitative relationship between EIS parameters and NaCl content is ultimately established. This work provides a theoretical and methodological basis for real-time monitoring of salt uptake in complex meat systems.

## 2. Materials and Methods

### 2.1. Raw Materials

Fresh *Longissimus dorsi* pork loins from Landrace pigs were purchased from a local supermarket in Zhenjiang, China. Muscle pH was measured at multiple sites using a calibrated direct insertion pH meter (PH5S, San-Xin Instrument Ltd., Shanghai, China), standardized with pH 4 and pH 7 buffers. Only samples with a pH value ranging from 5.5 to 5.7 were selected for subsequent experiments. Subcutaneous fat and connective tissue were carefully removed, and the meat was accurately cut into cuboid-shaped specimens (50 mm × 30 mm × 10 mm, 20 ± 2 g) using a sharp knife. The prepared samples were then stored at 6.8°C for further experimental analysis. NaCl, ammonium ferric sulfate, potassium thiocyanate, nitric acid, and silver nitrate were of analytical grade and purchased from Shanghai Sinopharm Reagent Co., Ltd., Shanghai, China.

### 2.2. Marination Treatments

Ultrasonic marination treatments were conducted using a custom-built ultrasound device developed by our research group. The detailed schematic diagram of this apparatus is presented in our previous study [4]. During the ultrasonic marination process, preliminary experiments demonstrated that the optimal curing effect was achieved at an output current of the ultrasonic generator of 0.15 A, corresponding to a nominal power of 33 W. Under this condition, the actual output power of the ultrasonic generator was 23.5 W. When the actual ultrasonic power was below 23.5 W, insufficient energy input resulted in an inconspicuous curing effect. Conversely, when the power exceeded 23.5 W, the rapid increase in temperature hindered the curing process due to thermal degradation. Therefore, in subsequent experiments, an ultrasonic power of 23.5 W was selected for ultrasonic-assisted marination. The acoustic energy density (AED) within the ultrasonic chamber was calculated based on the actual output power of the ultrasonic generator and remained constant at 0.43 W/mL throughout the experiment. The specific calculation formula is as follows:

(1) AED=P/V

where *P* is the actual output power of the ultrasonic generator (W), while *V* is the volume of the curing liquid added to the ultrasonic reactor chamber (mL).

Three slices of pork samples (approximately 60 g in total) were evenly positioned within the reaction chamber of an ultrasonic system (maximum capacity: 1000 mL; diameter: 11 cm) and treated at frequencies of 23.6, 26.8, 32.3, 40, and 55 kHz, respectively. Subsequently, a total of 55 mL of precooled curing solution (10% NaCl) was added to each chamber, which was then placed in a medical refrigerator maintained at 6.8 °C for curing. The curing durations were set at 5, 10, 15, 20, and 25 min. The muscle fiber orientation of the pork samples was aligned parallel to the direction of ultrasonic wave propagation. Temperature changes at the center of the meat samples during the ultrasonic curing process were monitored using a dual-channel K-type thermocouple thermometer. For the control group, pork samples were statically cured in the reaction chamber without ultrasonic treatment, while all other experimental conditions were kept identical. After the curing process, the samples were removed from the ultrasonic reaction chamber, and excess brine on the meat surface was absorbed with filter paper prior to further analysis.

### 2.3. Determination of NaCl Content

The NaCl content in pork was assessed using the Mohr titration method described in our previous study [16]. Briefly, five grams of the minced sample was carbonized and diluted with 100 mL of deionized water. Subsequently, 50 mL of the obtained solution was mixed with 5 mL of nitric acid solution and 25 mL of silver nitrate solution, and the mixture was kept in the dark for 5 min at room temperature. After filtration, 2 mL of ferric indicator solution was added, and the mixture was titrated with standard potassium thiocyanate solution (KSCN, 0.104 M) until a permanent orange-yellow color appeared.

### 2.4. Determination of Water Content

The minced sample (2 g) was placed into a pre-weighed weighing bottle and spread uniformly. The bottle was then placed in an oven set to 105 °C, with the cap slightly tilted against the edge to facilitate moisture evaporation. After 4 h of drying, the bottle was sealed and transferred from the oven to a desiccator for cooling before weighing. This procedure was repeated until the weight difference between two consecutive measurements was less than 2 mg. The final mass of the sample was recorded once a constant weight was achieved. The calculation formula is as follows:

(2) $$X=\frac{m_{1}-m_{2}}{m_{1}-m_{3}}\times 100$$

where *X* represents the moisture content of the sample (%); *m*_1_ is the combined mass of the weighing bottle and the sample (g); *m*_2_ is the combined mass of the weighing bottle and the dried sample (g); and *m*_3_ is the mass of the empty weighing bottle (g).

### 2.5. Simulation Analysis of NaCl Dynamics in Pork Tissue

Finite element simulations were performed using COMSOL Multiphysics ^®^ version 5.2 to investigate the mass transfer behavior within the meat matrix during marination. COMSOL Multiphysics is a multiphysics simulation software based on the finite element method, which solves partial differential equations through a graphical user interface (GUI). One major advantage of COMSOL Multiphysics is its user-friendly human–computer interaction, featuring simple and intuitive operations that help reduce the complexity of computer programming. Another advantage lies in its flexibility for modeling complex geometries and coupling multiple physical fields. In addition, COMSOL Multiphysics provides a variety of built-in post-processing tools, enabling convenient numerical visualization and analysis of simulation results. The simulation methodology was adapted with modifications from the approach described by Shi et al. [36]. To ensure computational efficiency and maintain model accuracy, the meat sample, with dimensions of 50 × 30 × 10 mm, was geometrically divided into eight equal segments. Among these, the segment closest to the surface exposed to ultrasonic treatment was selected as the representative modeling domain because it lies near the primary region of mass-transfer activity.

The transient diffusion process within the selected domain was governed by Fick’s second law, represented by the following partial differential equation:

(3) $$\frac{\partial c}{\partial t}+\nabla \cdot \left(-D\nabla c\right)=0$$

where *c* denotes the concentration of solute (marinade component) at time *t*, and *D* represents the diffusion coefficient.

Two types of boundary conditions were applied in the simulation. The three surfaces of the modeling domain that were in direct contact with the marinade solution were subjected to Dirichlet boundary conditions, defined as

*c* = *c*_0_ (4)

where *c*_0_ is the initial concentration of the marinade at the boundary.

All remaining surfaces, which were not in contact with the marinade, were treated as flux-free (Neumann boundary conditions), mathematically expressed as

(5) $$\left(-D\nabla c\right)\cdot n=0$$

where *n* is the outward unit normal vector to the surface, indicating that no solute flux occurs across these boundaries.

### 2.6. Thermogravimetric Analysis

The thermal stability of samples was evaluated using a TG/DTA analyzer (STA 499C, Netzsch Gerätebau, Selb, Germany) following the method of [37] with slight modification. Specifically, a 10 mg sample was placed in an alumina crucible and heated from 30 to 600 °C at a rate of 10 °C/min under a nitrogen flow of 50 mL/min. An empty sealed aluminum crucible was used as the reference.

### 2.7. Measurement of Electrical Impedance Spectroscopy

**Figure 1:**
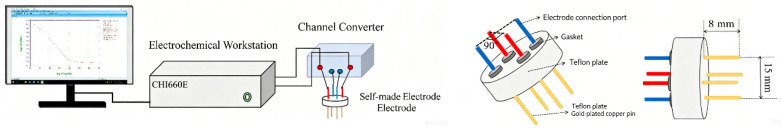
Schematic diagrams of the impedance acquisition system and the self-made electrode.

An impedance spectrum acquisition system was employed to collect the electrical impedance spectra of fresh and cured pork slices at room temperature. The system consisted of an electrochemical workstation (CHI660E, CH Instruments, Inc., Austin, TX, USA), a computer equipped with CHI660E impedance acquisition software(Ver. 16. 07), a channel converter, and a custom-designed electrode sensor featuring gold-plated copper needle electrodes, as shown in Figure 1. The detailed description of the self-made electrode can be found in the work of Sun et al. [38]. Two electrodes were vertically inserted into the pork slice to a depth of 8 mm, maintaining a 15 mm spacing between them. The current flow direction of the electrodes was parallel to the myofibril orientation. Once the electrodes were securely positioned, the impedance spectrum of the sample was recorded. The frequency range for testing was set from 0.1 Hz to 10^5^ Hz, with 73 discrete frequency points collected for each impedance spectrum. Each experimental group underwent nine replicate tests to ensure data reliability.

### 2.8. Analysis of Equivalent Circuit

Biological tissue was modeled as an ionized liquid medium, comprising uniformly distributed cells along with their liquid components, membranes, intracellular fluid, and extracellular fluid. The Fricke model, a method that simulates biological systems at the microscopic level using electronic components, has been widely applied in impedance analysis. However, due to the irregular size and shape of muscle tissue cells and the variability of cell membrane properties influenced by capacitance, the standard Fricke model cannot accurately fit experimental results. To more precisely reflect the true impedance of muscle tissue, a modified Fricke model was applied [39]. The electrical impedance spectral data of fresh pork and cured pork tissue were imported into ZView 3.1 software (Scribner Associates Inc., Southern Pines, NC, USA) and analyzed using the modified Fricke model. This process yielded key structural circuit parameters, including the intracellular fluid resistance (R_i_), extracellular fluid resistance (R_e_), and cell membrane capacitance (*C_m_*). These parameters were further examined to evaluate the changes in electrical impedance characteristics induced by ultrasonic curing.

### 2.9. Statistical Analysis

The experimental results were analyzed using one-way analysis of variance (ANOVA) in SPSS 19.0 software (SPSS Inc., Chicago, IL, USA), with Duncan’s multiple-range test used to determine significant differences between data groups (*p* < 0.05). Each experiment was repeated at least three times, and the results were expressed as the mean ± standard deviation (SD). Figures and graphs were generated using Origin 2018 C software (Origin Lab., Northampton, MA, USA). Curve regression was applied to evaluate the relationships among impedance magnitude, phase angle, and NaCl content.

## 3. Results and Discussion

### 3.1. Effects of Ultrasonic Frequencies on Water Content in Pork

There are two primary mass transfer processes during the marination process: the migration of water from muscle tissue to the marinade and the diffusion of NaCl from the marinade into the muscle tissue, which together facilitate the redistribution of NaCl and water [31]. The transfer dynamics of NaCl content have been investigated in our previous research [16]. In this study, the effects of conventional marination and ultrasonic marination at various frequencies on the moisture content of pork were evaluated, as shown in Table 1. The moisture content in pork samples across all groups exhibited an upward trend with prolonged marinating duration. However, the differences were not statistically significant (*p* > 0.05). Similarly, there were no significant variations in moisture content among the samples within each group (*p* > 0.05). These findings suggested that short-term marination did not markedly enhance the diffusion of moisture from the marinade into pork tissue, which was consistent with the conclusions of Zhao et al. [40]. However, the moisture content of pork exhibited a slight increase toward the end of the marination process. It was attributed to the fact that NaCl gradually infiltrated the pork tissue, partially dissolving salt-soluble myofibrillar proteins under prolonged marinating duration. Meanwhile, electrostatic repulsion among myofibrillar filaments induced myofibril expansion and generated internal expansion pressure [41]. When this pressure surpassed the osmotic pressure generated by the marinade, the muscle tissue gained a certain capacity to absorb water [42]. Consequently, the moisture content of pork exhibited a slight increase toward the end of the marination process.

### 3.2. Multiphysics Simulation of NaCl Diffusion in Pork

**Figure 2:**
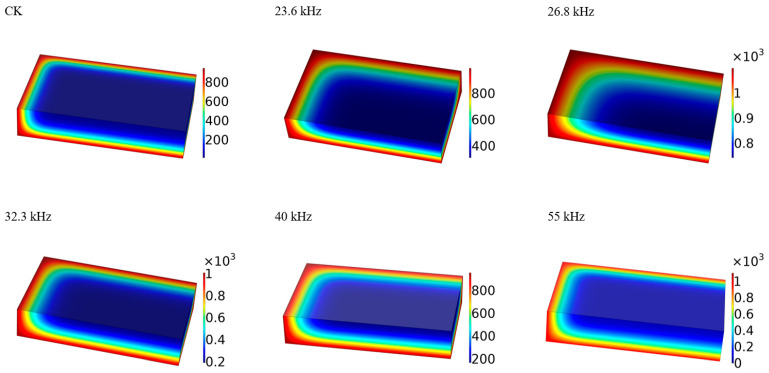
Simulation of NaCl penetration in pork samples subjected to various ultrasonic frequencies after 25 min of marination.

Figure 2 presents the penetration profiles of NaCl in pork samples subjected to different ultrasonic frequencies after 25 min of marination, providing a clear and intuitive visualization of the diffusion behavior during the marination process. From the perspective of color distribution in Figure 2, the edge area of the sample appears red and gradually transitions to yellow, green, and finally blue towards the interior, representing that the NaCl content decreases gradually from the outside to the inside. This phenomenon reflects the typical gradient feature of salt diffusion from the surface to the interior during the marination process. That is, the outer layer is first enriched with salt, while the inner area, due to the longer diffusion distance, has a relatively lower NaCl content. It can be seen that all ultrasonic treatments significantly enhanced NaCl penetration depth compared to CK. Notably, the greatest penetration depth and diffusion area were observed at 26.8 kHz, suggesting that ultrasound at this moderate frequency most effectively facilitated solute diffusion. This enhancement can be attributed to intensified cavitation effects, in which the formation and collapse of cavitation bubbles generated more microchannels within the tissue, promoting diffusion. However, when the frequency was further increased to 55 kHz, the penetration depth and uniformity declined. This may be due to greater energy attenuation at higher frequencies, which weakened the cavitation effect and reduced its ability to enhance diffusion [19]. Overall, ultrasonic frequency has a significant impact on NaCl diffusion in pork tissue, with 26.8 kHz appearing to be an optimal frequency for achieving efficient marination, which was consistent with our previous results [16].

### 3.3. Validation of Multiphysics Model Reliability

Table 2 presents the NaCl content in pork under these optimized ultrasonic parameters, as well as under conventional (non-ultrasonic) marination conditions using different brine concentrations (10%, 12%, and 15%). It can be observed that with a 10% brine solution, ultrasonic marination resulted in an NaCl content of 0.97% in pork. In contrast, conventional marination with the same 10% solution resulted in a lower NaCl content of only 0.70%. Notably, only when the brine concentration was increased to 12% in conventional marination did the NaCl content in pork approximate the level achieved through ultrasonic marination using a 10% solution over the same duration. These findings clearly demonstrated that ultrasonic marination enhanced salt penetration efficiency, thereby allowing for a reduction in brine concentration without compromising the final NaCl content in the pork. This suggested a promising potential for ultrasonic technology to improve the efficiency and sustainability of the pickling process.

**Figure 3:**
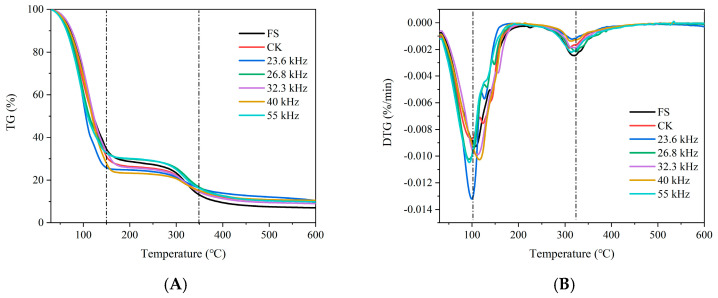
Thermal stability of pork under different ultrasonic frequency treatments: TG (**A**) and DTG (**B**) analyses. FS: fresh pork samples; CK: marinated samples without ultrasound treatment.

Thermogravimetric analysis was employed to characterize pork samples marinated for 25 min under different ultrasonic frequencies in order to validate the reliability of the multiphysics model. The quality loss of the temperature function of pork meat at 30–600 °C was investigated, as shown in Figure 3. It can be seen that TG curves were roughly divided into three stages, including the rapid water evaporation stage, the thermal decomposition of organic substances stage, and the slow degradation stage. The first range of 30–150 °C caused rapid water degradation, including bound water, intramolecular water, and free water, mainly due to water being the primary content in pork meat. In detail, the evaporation rate of water varied slightly for different frequencies. It can be observed that the residual mass in the ultrasonic treatment groups decreased more rapidly at this stage, suggesting that ultrasound may enhance the migration and release of water within tissues, thereby facilitating evaporation during heating. The second range of 150–350 °C was the main cause for the thermal decomposition of organic substances (such as proteins and fats), leading to a sharp drop in the slope of the curve. The third range of 350–600 °C caused the pyrolysis residues to gradually carbonize, and the changes tended to be gentle. All samples tended to stabilize at this stage, indicating that the major mass loss was complete. At the time, the residual quality of fresh pork was significantly lower than that of the other pickled groups. This was mainly because NaCl permeated into the pork tissue during the pickling process, and NaCl would not volatilize or decompose within this temperature range, thus remaining in the sample. Figure 3B shows the DTG curves that were the first derivative of the quality loss curve, representing the degradation rate. It was observed that the first inflection point was near 100 °C, corresponding to the first stage of the TG curves. The second inflection point occurred near 315 °C, which was caused by the degradation of protein, fats, and other organic substances.

Overall, under ultrasonic marinating conditions with a frequency of 26.8 kHz, an ultrasonic power of 23.5 W, a marinade volume of 55 mL, and a curing time of 25 min, the NaCl content in pork reached its highest level within the consumer-acceptable range.

### 3.4. Effects of Marinating Duration on Equivalent Circuit Parameters of Pork Tissue

**Figure 4:**
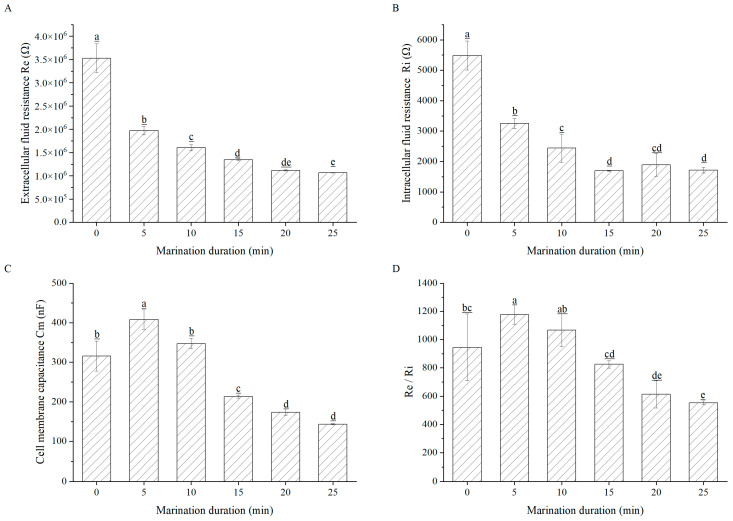
Extracellular fluid resistance (**A**), intracellular fluid resistance (**B**), cell membrane capacitance (**C**), and the ratio of extracellular fluid resistance to intracellular fluid resistance (**D**) of pork under different marination times with an ultrasonic frequency of 26.8 kHz. Different letters in the same row indicate a statistically significant difference (*p* < 0.05).

The changes in electrical characteristics of pork tissues induced by NaCl diffusion, facilitated by ultrasonic treatment at 28 kHz during the marination process, were assessed through equivalent circuit parameters using a modified Fricke model. As shown in Figure 4, both extracellular fluid resistance (R_e_) and intracellular fluid resistance (R_i_) in pork cells gradually decreased as the pickling time progressed. The capacitance of pork cell membrane (*C_m_*) and the resistance ratio of extracellular fluid to intracellular fluid (R_e_/R_i_) initially increased and subsequently decreased with pickling time. In detail, all parameters of electrical components underwent significant changes within the first 15 min of marination, while the variation trend stabilized between 15 and 25 min. At the early stage of marination, a substantial pressure difference existed between the brine solution and the extracellular fluid of pork cells, which drove NaCl to continuously penetrate the meat tissue. This process initially increased the ionic strength of the extracellular fluid, thereby reducing its electrical resistance. Subsequently, as NaCl accumulated within the pork, it promoted the partial dissolution of myofibrillar proteins and allowed some NaCl to enter the intracellular fluid, ultimately reducing its resistance. Additionally, within the first 0–5 min of marination, *C_m_* increased, potentially due to the reduction in phospholipid bilayer thickness or an increase in membrane surface area induced by NaCl [43]. After 5 min, progressive cell membrane damage led to a decline in membrane capacitance. Simultaneously, NaCl induced the rotation of protein dipoles within muscle tissue, contributing to energy dissipation and further weakening the capacitive properties. R_e_/R_i_ served as an indicator of cell membrane permeability, where a lower ratio signifies greater permeability [44]. As shown in Figure 4D, R_e_/R_i_ decreased with prolonged marination, indicating a gradual enhancement of membrane permeability throughout the process.

### 3.5. Effects of Marinating Duration on Electrical Impedance Characteristics of Pork Tissue

**Figure 5:**
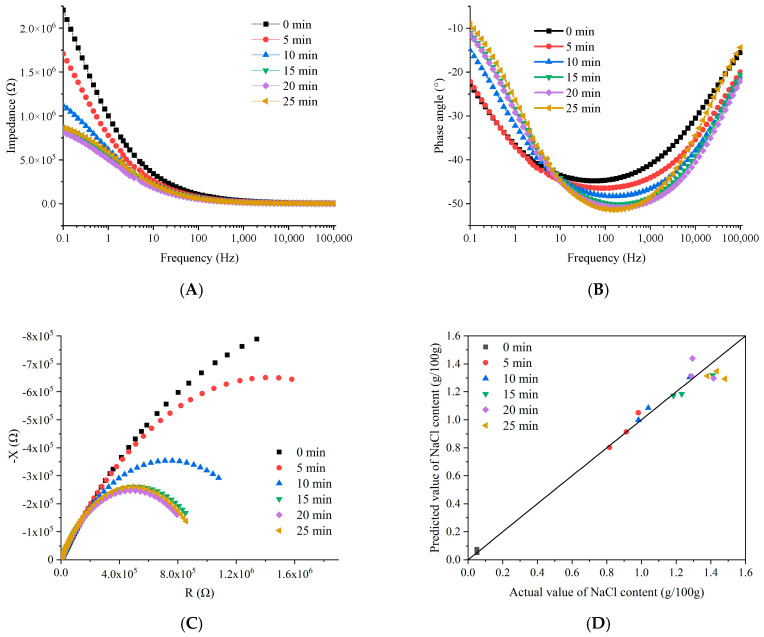
Electrochemical characteristics of pork samples marinated at 26.8 kHz for different durations. Frequency-impedance plot (**A**), frequency-phase angle plot (**B**), Cole–Cole plot (**C**), and scatter plot of observed vs. predicted NaCl content in pork (**D**).

The variation in impedance of pork tissue with excitation current frequency under different marinating durations at an ultrasonic frequency of 28 kHz is presented in Figure 5A. It was observed that the impedance of all samples decreased with increasing current frequency. This trend was particularly pronounced at frequencies below 1000 Hz, where impedance exhibited a strong dependence on frequency. At a given frequency, the non-marinated sample showed the highest impedance value. As the marinating duration increased, the impedance of the samples gradually decreased. However, no significant differences in impedance were observed among samples marinated for 15, 20, and 25 min. Due to the anisotropic nature of muscle tissue, impedance varies with the direction of current flow through the tissue [45]. This anisotropy is not only related to the arrangement of muscle fibers but also closely associated with the distribution and migration of ions within the tissue [46]. During the marination process, prolonged marinating time led to a gradual increase in ion concentration (particularly Na^+^ and Cl^−^) in the muscle tissue, which enhanced electrical conductivity and consequently reduced impedance. These findings demonstrated that marinating time significantly influenced the electrical properties of pork tissue by modulating ion concentration, which was crucial for the precise prediction and control of pork marination processes using electrical impedance measurements. Furthermore, when the excitation current frequency exceeded 1000 Hz, the impedance remained relatively stable, indicating a diminished dependence on the current frequency. It was attributed to cell membranes losing their insulating properties at high frequencies, leading to current flowing through the membranes into the intracellular fluid. Conversely, the cell membrane had a high capacitance at low frequencies, and the current could only be conducted in the extracellular fluid [47].

As depicted in Figure 5B, the phase angle of pork samples initially increased and subsequently decreased with increasing current frequency, forming a saddle-shaped curve. Within the range of increasing current frequency, the phase angle variation in non-marinated pork was smaller compared to that of marinated pork. Additionally, the amplitude of phase angle variation became more remarkable as the marinating duration increased. However, after 15 min of marination, this variation began to level off. In non-marinated pork, the cell structure was intact with Na^+^ and Cl^−^ predominantly present in the extracellular fluid, K^+^ serving as the primary cation in the intracellular fluid, and phosphate and protein acting as the main intracellular anions. As a result, the extracellular and intracellular fluids can be regarded as electrolytes, while the cell membrane functions as a capacitor. The electrochemical properties are primarily evaluated based on the mobility of ions involved in their metabolic processes [48,49]. When a low-frequency current was applied to an unpickled sample, the high impedance of the cell membrane prevented most of the current from penetrating it, resulting in impedance being predominantly determined by the resistance of the extracellular fluid. This led to a large total impedance amplitude and a small phase angle. Conversely, when a high-frequency current passed through the sample, the capacitive reactance of the cell membrane gradually decreased, allowing the current to traverse both the extracellular and intracellular fluids [29]. As a result, the total impedance value diminished, and the phase angle increased progressively. However, when the current frequency continued to rise, the cell membrane effectively acted as a conductive pathway, causing the phase angle to decrease once again [38,50]. Following marination in a NaCl solution, the impedance of pork samples decreased, while the phase angle increased. This was primarily due to the continuous diffusion of NaCl into the muscle tissue during curing, which elevated the ion concentration within the cells and subsequently enhanced the electrical conductivity of the pork tissue.

The typical Cole–Cole plots of pork tissue samples with various marination durations are shown in Figure 5C. It was observed that the plots were semicircular arcs, confirming Cole’s theoretical models of electrical activity and correlating with impedance patterns characteristic of biological tissues [48]. The results showed that the uncured sample exhibited a large arc radius, indicating intact pork cells with an enclosed cellular structure. As the marinating time increased, the NaCl content in the pork gradually rose, and the arc radius of the samples progressively decreased [51]. observed that the Cole–Cole arc radius diminished with prolonged storage time of pork, and similar findings were reported in the curing process of beef [52], which was attributed to cell membrane damage [53]. Therefore, the findings of this study suggested that the cell membranes of pork tissue were compromised during marination, and longer curing times led to a smaller arc size, demonstrating that NaCl caused significant damage to the cell membranes.

### 3.6. Predicting the Content of NaCl in Pork

As elaborated in the above section, the increase in NaCl content in pork tissue induced substantial alterations in its electrochemical properties. Consequently, seven frequency points (0.1, 1, 10, 100, 1000, 10,000, and 100,000 Hz) were selected within the spectral range to investigate the relationship between the impedance values, phase angles, and NaCl concentration in pork. Table 3 and Table 4 present the impedance values and phase angles of pork at specific frequencies under varying marinating durations. It can be observed that at the selected frequencies, the impedance value exhibits a significant decreasing trend with the increase in marinating time. After 15 min, the impedance amplitude fluctuated within a defined range, while the phase angle did not display a similar pattern of variation.

A regression analysis model was constructed to explore the relationship between NaCl content in pork and impedance values, as well as phase angles at different marinating times. In this model, NaCl content was taken as the dependent variable, while impedance values and phase angles at a given current frequency were treated as independent variables, respectively. Table 5 presents the curve-fitting equations and correlation coefficients for the relationship between NaCl content and electrochemical parameters. It can be seen that impedance values at different current frequencies exhibited a strong linear correlation with NaCl content, with correlation coefficients (*R*^2^) ranging from 0.882 to 0.952. By contrast, the regression fit between phase angles and NaCl content at various current frequencies was less satisfactory. This may be attributed to the reduced sensitivity of phase angle responses to changes in NaCl content, which is influenced by the complex dielectric behavior and heterogeneous structure of pork tissue. In addition, factors such as electrode polarization reduction, intracellular current conduction, and tissue structural variability may further weaken the correlation between phase angle and NaCl content at these frequencies, resulting in poorer fitting performance. These results indicated that impedance values can effectively predict the variation in NaCl content during the pork curing process. Additionally, electrochemical impedance spectroscopy technology has been successfully applied to predict salt content during the salting and smoking of salmon [54], the curing of ham [23], and beef marination [52]. Electrical conductivity is considered to be related to the mobility of ions and the capacitance that includes muscle tissue information. During the marination process, NaCl gradually diffuses into the muscle, enhancing the tissue’s conductivity. The impedance modulus usually reflects changes in conductivity, establishing a strong correlation between impedance parameters and NaCl content [25].

To visually illustrate the predictive capability of impedance modulus for NaCl content in pork, a scatter plot was constructed, with the actual NaCl content determined by chemical methods plotted on the *X*-axis and the NaCl content predicted using the regression equation at 1000 Hz on the *Y*-axis, as depicted in Figure 5D. The plot revealed that both the actual and predicted values were closely aligned along the diagonal line, suggesting that impedance values can reliably predict NaCl content during the pork marination process.

## 4. Conclusions

This study demonstrated that short-term ultrasonic marination did not markedly enhance the diffusion of moisture from the pickling solution into pork tissue. However, simulation-based visualization of NaCl diffusion revealed that ultrasonic treatment promoted faster salt penetration, enabling a reduction in brine concentration without compromising the final NaCl content in the pork. Thermogravimetric (TG) analysis validated the simulation results, further confirming that ultrasound effectively accelerated NaCl penetration into meat tissue, as evidenced by the higher residual NaCl content and increased residual mass in ultrasound-treated samples. Furthermore, the electrical impedance characteristics of pork exhibited significant changes with increasing ultrasonic pickling time, including decreased impedance, an expanded phase angle, and a reduced radius of the Cole–Cole arc. These changes indicate the gradual infiltration of NaCl into the pork tissue, leading to enhanced electrical conductivity and structural modifications in muscle fibers. Moreover, a strong linear correlation was established between impedance values and NaCl content. Validation experiments confirmed that impedance measurements can accurately predict NaCl content during the marination process. Overall, this study provides a theoretical foundation and technical support for the non-destructive and real-time monitoring of NaCl content during meat marination, highlighting the promising application prospects of this technique.

## Figures and Tables

**Table 1:** Effect of different ultrasonic frequencies on the moisture content of marinated pork.

Marination Time (min)	Ultrasonic Frequency (kHz)					
	CK	23.6	26.8	32.3	40	55
5	72.70 ± 0.40 a	72.35 ± 1.23 a	71.58 ± 0.83 a	71.85 ± 1.53 a	72.43 ± 0.99 a	72.02 ± 0.97 a
10	72.70 ± 0.54 a	73.62 ± 0.56 a	71.72 ± 1.16 a	72.08 ± 1.30 a	72.47 ± 0.19 a	72.10 ± 0.21 a
15	72.82 ± 0.77 a	72.76 ± 0.58 a	71.81 ± 0.43 a	72.42 ± 0.67 a	72.47 ± 0.10 a	72.43 ± 0.51 a
20	72.91 ± 0.54 a	72.97 ± 1.32 a	72.17 ± 0.60 a	72.85 ± 0.53 a	72.67 ± 0.10 a	72.97 ± 0.21 a
25	72.97 ± 0.16 a	73.57 ± 0.52 a	73.28 ± 1.07 a	73.61 ± 0.31 a	73.38 ± 0.29 a	73.39 ± 0.23 a

Note: CK: marinated samples without ultrasound treatment. Moisture content values are expressed as mean ± SD (%, *w*/*w*). Different letters in the same row indicate a statistically significant difference (*p* < 0.05). There was no significant difference in moisture content among the groups (*p* > 0.05).

**Table 2:** Effects of marination methods and saline solution on NaCl content in pork.

Saline Solution	26.8 kHz	CK		
	10%	10%	12%	15%
NaCl in pork (%)	0.97 ± 0.15	0.70 ± 0.05	0.96 ± 0.09	1.21 ± 0.10

**Table 3:** Impedance (Z) of pork at given current frequencies under different marination times with an ultrasonic frequency of 26.8 kHz.

Marination Time (min)	Current Frequency (Hz)						
	0.1	1	10	100	1000	10,000	100,000
0	2205.10 ± 154.05 a	1000.90 ± 93.05 a	343.83 ± 34.38 a	109.45 ± 10.95 a	34.93 ± 3.49 a	13.38 ± 1.82 a	7.46 ± 0.92 a
5	1707.40 ± 170.74 b	778.64 ± 77.86 b	261.42 ± 26.14 b	80.24 ± 8.02 b	24.18 ± 2.42 b	8.35 ± 0.84 b	4.04 ± 0.40 b
10	1118.40 ± 111.84 c	616.78 ± 61.68 c	218.90 ± 21.89 bc	65.15 ± 6.52 c	18.50 ± 1.85 c	5.94 ± 0.59 c	2.72 ± 0.27 cd
15	868.19 ± 86.82 d	546.40 ± 54.82 c	204.63 ± 20.46 bc	59.11 ± 5.91 c	15.91 ± 1.59 cd	4.87 ± 0.49 cd	2.23 ± 0.22 de
20	811.58 ± 81.16 d	502.56 ± 50.26 c	183.75 ± 18.38 c	52.13 ± 9.16 c	13.77 ± 1.38 d	4.10 ± 0.41 d	1.79 ± 0.18 e
25	867.56 ± 86.76 d	584.42 ± 58.44 c	223.11 ± 22.36 c	62.05 ± 6.21 c	16.14 ± 1.61 cd	5.19 ± 0.52 cd	2.84 ± 0.28 c

Note: All impedance values are in kilohms (kΩ). Different letters for values in the same row indicated a statistically significant difference (*p* < 0.05).

**Table 4:** Phase angle (θ) of pork at given current frequencies under different marination times with an ultrasonic frequency of 26.8 kHz.

Marination Time (min)	Current Frequency (Hz)						
	0.1	1	10	100	1000	10,000	100,000
0	−22.83 ± 2.28 c	−36.66 ± 3.67 b	−43.51 ± 4.35 a	−44.67 ± 4.47 a	−41.02 ± 4.10 a	−30.48 ± 3.05 a	−15.55 ± 1.56 a
5	−22.21 ± 2.22 c	−37.07 ± 3.71 b	−44.70 ± 4.47 a	−46.47 ± 4.65 a	−44.08 ± 4.41 a	−35.39 ± 3.54 ab	−20.02 ± 2.00 b
10	−15.11 ± 1.51 b	−32.26 ± 3.23 ab	−44.40 ± 4.44 a	−48.22 ± 4.82 a	−46.60 ± 4.66 a	−37.92 ± 3.79 b	−21.21 ± 2.12 b
15	−11.07 ± 1.11 a	−28.36 ± 2.84 a	−44.15 ± 4.42 a	−49.84 ± 4.98 a	−48.57 ± 4.86 a	−39.03 ± 3.90 b	−20.56 ± 2.06 b
20	−11.42 ± 1.14 a	−29.20 ± 2.92 a	−44.97 ± 4.50 a	−50.54 ± 5.05 a	−49.47 ± 4.95 a	−40.50 ± 4.05 b	−22.00 ± 2.20 b
25	−9.12 ± 0.81 a	−26.41 ± 2.64 a	−44.69 ± 4.47 a	−51.24 ± 5.12 a	−48.50 ± 4.85 a	−34.49 ± 3.45 ab	−14.40 ± 1.44 a

Note: All phase angle values are in degrees (°). Different letters for values in the same row indicated a statistically significant difference (*p* < 0.05).

**Table 5:** Regression analysis of NaCl content in pork and electrical parameters (impedance and phase angle) in pork.

Current Frequency (Hz)	Curvilinear Regression Model	*R* ^2^
0.1	y = −0.001 Z + 2.096 y = −0.004 θ^2^ − 0.062 θ + 1.155	0.910 0.759
1	y = −0.003 Z + 2.78 y = −0.004 θ^2^ − 0.192 θ − 0.538	0.905 0.670
10	y = −0.008 Z + 3.048 y = −0.793 θ − 34.177	0.882 0.644
100	y = −0.024 Z + 2.711 y = −0.035 θ^2^ − 3.582 θ − 89.333	0.927 0.968
1000	y = −0.063 Z + 2.321 y = −0.018 θ^2^ − 1.824 θ − 43.659	0.950 0.977
10,000	y = −0.145 Z + 2.024 y = −0.019 θ^2^ − 1.464 θ − 26.776	0.952 0.781
100,000	y = −0.238 Z + 1.850 y = 0.061 θ^2^ + 2.165 θ + 19.445	0.915 0.425

## Data Availability

The original contributions presented in this study are included in the article. Further inquiries can be directed to the corresponding author.
